# Fine mapping of a dominant gene conferring chlorophyll-deficiency in *Brassica napus*

**DOI:** 10.1038/srep31419

**Published:** 2016-08-10

**Authors:** Yankun Wang, Yongjun He, Mao Yang, Jianbo He, Pan Xu, Mingquan Shao, Pu Chu, Rongzhan Guan

**Affiliations:** 1 State Key Laboratory of Crop Genetics and Germplasm Enhancement, Nanjing Agricultural University, Nanjing 210095, China; Jiangsu Collaborative Innovation Center for Modern Crop Production, Nanjing, Jiangsu, China; 2 Soybean Research Institute, Nanjing Agricultural University, Nanjing 210095, Jiangsu, China

## Abstract

Leaf colour regulation is important in photosynthesis and dry material production. Most of the reported chlorophyll-deficient loci are recessive. The dominant locus is rarely reported, although it may be more important than the recessive locus in the regulation of photosynthesis efficiency. During the present study, we mapped a chlorophyll-deficient dominant locus (*CDE1*) from the ethyl methanesulfonate-mutagenized *Brassica napus* line NJ7982. Using an F_2_ population derived from the chlorophyll-deficient mutant (*cde1*) and the canola variety ‘zhongshuang11’, a high-density linkage map was constructed, consisting of 19 linkage groups with 2,878 bins containing 13,347 SNP markers, with a total linkage map length of 1,968.6 cM. Next, the *CDE1* locus was mapped in a 0.9-cM interval of chromosome C08 of *B. napus*, co-segregating with nine SNP markers. In the following fine-mapping of the gene using the inherited F_2:3_ populations of 620 individuals, the locus was identified in an interval with a length of 311 kb. A bioinformatics analysis revealed that the mapping interval contained 22 genes. These results produced a good foundation for continued research on the dominant locus involved in chlorophyll content regulation.

Leaf colour mutations such as albinism, light green, yellowish, stripe fleck, and others have been identified in many plants^1^. Using mutant plants, researchers have shown that leaf colour changes may alter chloroplast ultrastructure, leaf physiological and biochemical characteristics, and genetic mechanisms regulating leaf colour. These changes usually lead to reduced chlorophyll (Chls a and b) content in leaf colour mutants. Physiologically, Chl plays an important role in harvesting light and transferring energy. It is involved in chloroplast development, photomorphogenesis, and signal transduction between the nucleus and chloroplast. Thus, leaf colour plays important roles in regulation of photosynthetic efficiency.

Many explanations for leaf colour variations have been provided. One reason for low Chl content in many higher plants is Chl biosynthetic deficiency^2^,^3^,^4^,^5^. Studies on the Chl biosynthetic process have focused on *Arabidopsis thaliana* and rice. Previous reviews and studies involving these two species revealed that at least 27 genes controlling 15 steps were involved in Chl biosynthesis from glutamyl-tRNA to Chls a and b^6^. Other reasons for Chl deficiency include deficient signal transduction between the nucleus and chloroplast^3^,^7^, restrained heme feedback^8^,^9^, impaired synthesis and importation of chloroplast proteins^10^,^11^,^12^,^13^,^14^,^15^, and harmful photooxidation^16^,^17^,^18^,^19^. Overall, the molecular mechanisms regulating leaf colour phenotypes are very complex.

Cytological, physiological, and proteomic studies on leaf colour mutations have been reported for *Brassica napus*^20^,^21^,^22^,^23^,^24^,^25^,^26^. A genetic analysis of a leaf colour mutation, *Cr*, in *B. napus* was first reported in a Chl-deficient mutant with a yellowish-leaf phenotype controlled by one recessive gene. This yellowish mutant had been introduced into male sterile lines and used as a marker to produce hybrid seed^26^. Another Chl-deficient mutant, *BnaC.ygl*, was controlled by one recessive gene mapped onto *B. napus* chromosome C06. The marker linked to it was within 0.03 cM^27^. A spontaneous yellowish-leaf mutant of *B. napus*, *BnChd1*, was controlled by two pairs of recessive genes. Its gene (or locus), *BnChd1-1*, was inferred to be Bra040517 located on an A01 chromosomal region based on functional and expression analyses of genes within the mapping region^28^.

We herein report the inheritance of a dominant Chl-deficient mutant (*cde1*) isolated from an ethyl methanesulfonate (EMS)-mutagenized *B. napus* line, as well as the gene mapping, together with a saturated single nucleotide polymorphism (SNP) linkage map. Our findings offer new insights into the dominant yellowish-leaf phenotype.

## Results

### Chl content

**Figure 1:**
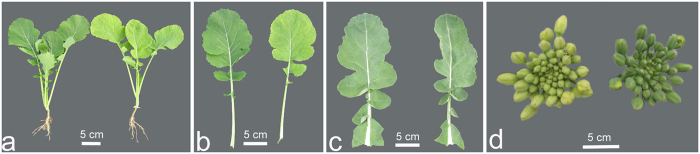
Morphological performance of the *cde1* mutant and its wild-type. (**a**) Wild-type plant (left) and *cde1* mutant plant (right) at the 6-week-old stage. (**b**) Leaves of the Wild-type (left) and *cde1* mutant plant (right). (**c**) The leaves of the *cde1* mutant plant (left) and Wild-type plant (right) after the budding stage. (**d**) The buds of the *cde1* mutant plant (left) and Wild-type plant (right).

Seedlings of the *cde1* mutant have yellowish leaves that gradually become green at the budding stage (Fig. 1a–c). The buds of the *cde1* mutant are light-yellow, whereas those of wild-type plants are green (Fig. 1d). The Chl content of *cde1* mutants and wild-type plants are provided in Table 1. The mutants had significantly lower Chls a and b and total Chl content, and a lower Chl a/b ratio than the wild-type plants.

### The influence of leaf colour on yield traits

The relationship of leaf colour with agronomic traits was investigated by randomly sampling the mature plants from Chl-deficient and green seedlings in the F_2:3_ populations. The results revealed that the 1,000-seed weight and yield of individual Chl-deficient plants were lower than that of normal green plants although many of the other traits were not significantly different between the two groups (Table 2). This clearly indicated that the yellowish leaves at the seedling stage reduced the yields.

### Inheritance of the yellowish-leaf trait

A cross between the canola variety ‘zhongshuang11’ (‘ZS11’) and our mutant *cde1* generated F_1_ plants with the same yellowish leaves as the mutant. By selfing the F_1_ plants, we generated 157 F_2_ plants that consisted of 119 yellowish-leaf and 38 green-leaf plants. Results of a chi-squared test indicated that the segregation pattern agreed with the Mendelian segregation ratio of 3:1 (yellowish-leaf vs. green-leaf plants) (Table 3). Thus, the yellowish-leaf trait is controlled by a single dominant gene. Additionally, results for the F_2:3_ family populations from the four selfed heterozygous plants also agreed with an expected Mendelian inheritance ratio of 3:1 (yellowish-leaf vs. green-leaf plants) (Table 3). This further demonstrated that the yellowish-leaf trait was controlled by one dominant gene. The dominant inheritance of this Chl deficiency phenotype is rare in nature and differs from the inheritance reported for most previously described yellowish-leaf mutants^23^,^24^,^26^,^27^,^28^.

### Construction of a high density SNP map

From the (‘ZS11’ × *cde1*) F_2_ population, 89 plants were used for SNP genotyping. Although the chip has 52,157 SNP markers, the 14,377 polymorphic markers were used to construct the linkage map after removing invalid markers. The 19 linkage groups (LGs) produced by the JoinMap 4 software contained 2,878 bins representing 13,347 SNP markers (Supplementary Data). The total length of the map was 1,968.6 cM. The longest LG was C03 at 173.9 cM and the shortest was C02 at only 41.8 cM. The mean interval between adjacent markers was 0.69 cM (Table 4). These data indicated that our linkage map based on SNP markers was likely sufficient to map the locus controlling the yellowish-leaf trait.

### Mapping the *CDE1* locus

**Figure 2:**
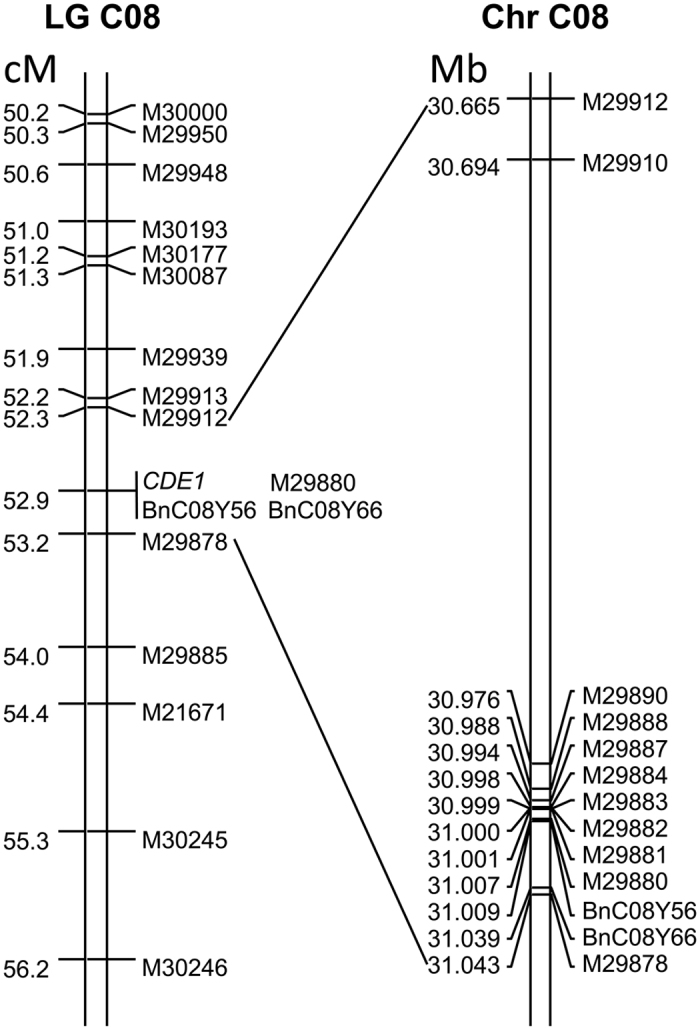
Mapping interval for *CDE1* on LG C08 and its corresponding physical map of chromosome C08 of *Brassica napus* cv. ‘ZS11’. This linkage map was a part of LG C08 in supplemental table S1, and the physical map was obtained by inquiries of ‘ZS11’ genome database with SNP probes.

By linking the leaf colour phenotypes with the corresponding SNP marker data for the F_2_ population, the yellowish-leaf trait locus was mapped onto LG C08, positioned in a 0.9 cM interval between SNP markers M29912 and M29878. The *CDE1* locus co-segregates with the bin M29880, which has eight co-segregated SNP markers: M29910, M29890, M29888, M29887, M29884, M29883, M29882, and M29881. By searching databases containing the genomes of *B. oleracea* and *B. napus* cv. ‘ZS11’, we found that probe sequences for the nine co-segregating SNPs completely matched genome sequences. In the *B. napus* cv. ‘ZS11’ genome (http://www.ncbi.nlm.nih.gov/assembly/GCA_000686985.1), the interval harboring the *CDE1* locus was 378.32 kb (Fig. 2).

**Figure 3:**
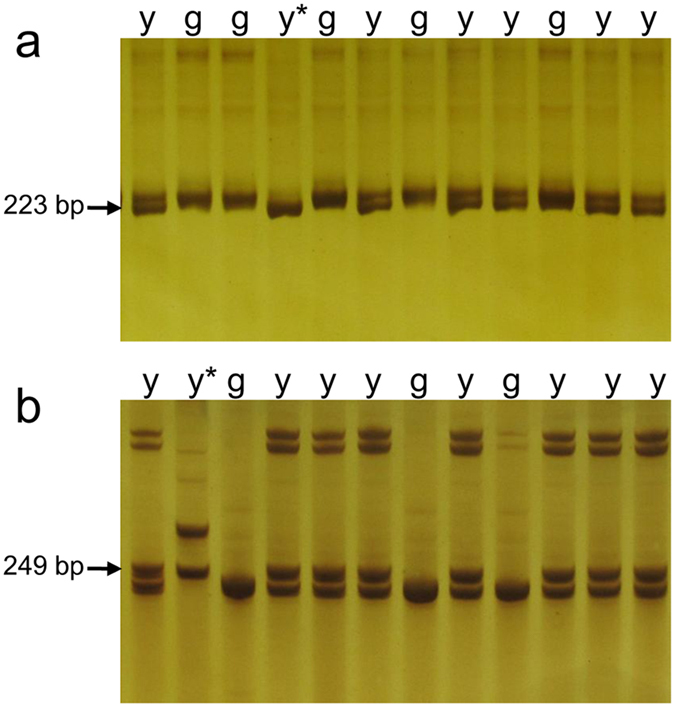
PCR products of SSR codominant markers BnC08Y56 and BnC08Y66 in the F_2_ population. Here, ‘y*’ and ‘g’ indicates products from homozygous yellowish-leaf plants and green-leaf plants, respectively. ‘y’ indicates a heterozygous yellow-leaf plant. (**a**,**b**) are the PCR products of SSR markers BnC08Y56 and BnC08Y66, respectively.

To narrow the mapping interval for further study, simple sequence repeat (SSR) markers uniformly covering the interval were developed based on the target interval genomic sequence. In total, 135 pairs of SSR primers were developed (Supplementary Table S1). These markers were used in the mapping population. However, only two pairs of primers, BnC08Y56 and BnC08Y66, were polymorphic and acted as co-dominant markers. These two SSR markers generated the expected products (Fig. 3) and co-segregated with the *CDE1* locus in the F_2_ population.

**Figure 4:**
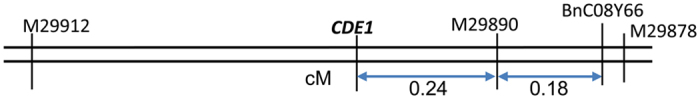
Fine mapping of *CDE1* on chromosome C08 of *Brassica napus* with SSR and SNP markers in the F_2:3_ populations.

We conducted additional experiments using 620 individuals of the F_2:3_ populations generated from the cross involving the tightly linked polymorphic markers BnC08Y66 and BnC08Y56. We obtained four recombinants between the *CDE1* locus and two SSR markers with green-leaf phenotype. The recombinants identified by this SSR marker experiment were confirmed by a SNP marker experiment that adopted the same technology as that used for the F_2_ plant genotyping. Furthermore, the SNP marker experiment demonstrated that of the four recombinants, two occurred between M29910 and M29890 and two occurred between M29888 and M29887. These data may help estimate the recombination rate. The distance between *CDE1* and SNP marker M29890 was 0.24 cM, and the distance between *CDE1* and SSR marker BnC08Y66 was 0.42 cM (Fig. 4). Therefore, the locus was on a 311-kb chromosomal interval between SNP markers M29912 and M29890 of the *B. napus* cv. ‘ZS11’ genome (http://www.ncbi.nlm.nih.gov/assembly/GCA_000686985.1).

### *Brassica* segments homologous to the mapped interval

An analysis of the homologous segments of the interval carrying the *CDE1* locus was necessary. The searches in *Brassica* genome databases including that of *B. rapa* (http://brassicadb.org/brad/), *B. oleracea* (http://brassicadb.org/brad/), *B. napus* cv. ‘Darmor-*bzh*’ (http://www. Genos cope.cns.fr/ brassicanapus/), and *B. napus* cv. ‘ZS11’ (http://www.ncbi.nlm.nih.gov/assembly/GCA_000686985.1) with the aid of SNP probes generated five homologous segments (Table 5).

Alignments among the homologous segments with dot matrix software (http://blast.ncbi.nlm.nih.gov/Blast.cgi), except that of *B. napus* cv. ‘Darmor-*bzh*’ that contains a large gap of approximately 70 kb, showed that the two C08 segments were structurally similar, with slight differences in the structure of the two A09 segments (Supplementary Fig. S1). However, the similarities between the two A09 segments were almost the same as those between the two C08 segments. These results were consistent with the evolutionary relatedness among *Brassica* species.

The genes of *B. rapa*, *B. oleracea*, and *B. napus* cv. ‘Darmor-*bzh*’ have been annotated. However, the target mapping interval of the genome of *B. napus* cv. ‘Darmor-*bzh*’ has genomic gap of 70 kb in length because of its imperfect genome assembly, leading to the absence of part of the gene annotation. Unfortunately, the genes in the genome of *B. napus* cv. ‘ZS11’ (one parent in our study) have not been annotated. This made it difficult to find candidate genes responsible for the yellowish-leaf trait. Nevertheless, the extensive information available regarding the *Brassica* genome annotation might be helpful. By use of the homologue search approach, we annotated the genes for the un-annotated segment, and replenished gene information for the annotated segments as shown in Table 5. Based on these works, the mapped *B. napus* cv. ‘ZS11’ segment was discovered to contain 22 genes, and the other homologous segments contained 17–23 genes (Table 5). Additionally, the gap in the C08 segment from ‘Darmor-*bzh*’ may be filled with a 77-kb segment from the unanchored “chrC08_random” pseudo-molecule, based on its homology to the other four segments (Supplementary Fig. S2). As a result, the C08 segment from ‘Darmor-*bzh*’ was discovered to contain 23 genes.

### Candidate genes analysis

Based on the gene codes on the homologous segments, as illustrated in Table 5, we inquired the genome databases of *Brassica* genus (http://brassicadb.org/brad/, http://www.genoscope.cns.fr/brassicanapus/, and http://www.ncbi.nlm.nih.gov/assembly/GCA_000686985.1) to find gene function information based on the putative function of their homologous genes in *A. thaliana* (Table 5). BnaC08g34740D, BnaC08g34800D, and BnaC08g34840D are directly involved in Chl biosynthesis. The gene BnaC08g34740D homologous to AT2G22880, encoding VQ motif-containing protein (VQ12), acting as a cofactor of WRKY transcription factors, may be the candidate gene responsible for the yellowish-leaf trait because the mutant *vq8-1* in *Arabidopsis* exhibits the yellowish leaf phenotype, whereas VQ8 is homologous to VQ12 and has an N-terminal signal peptide that is predicted to be chloroplast targeting^29^. BnaC08g34800D is homologous to AT1G78490 that encodes cytochrome P450 protein CYP708A3 with heme-binding activity, and may participate in Chl metabolism. BnaC08g34840D is homologous to AT5G4258, which encodes a cytochrome P450 protein CYP705A12 and has a similar function as CYP708A3. Thus, two genes, BnaC08g34800D and BnaC08g34840D may regulate leaf colour because of functional changes^30^. All the three genes may lead to recessive Chl-deficient phenotype, but they are far from being good candidate genes for *cde1* mutant to explain the dominance mechanism. Our DNA sequencing experiments showed that these three genes have no sequence differences between the *cde1* mutant and ‘ZS11’.

BnaC08g49180D and BnaC08g34850D are homologous to AT2G22760 and AT2G22750, respectively, both of which belong to the family of bHLH transcription factor genes. A member of the bHLH transcription factor family, PIF3 (Phytochrome-Interacting Factor 3), is a direct phytochrome reaction partner in the photoreceptor’s signalling network^31^. Besides PIF3, bHLH transcription factor family members PIF1, PIF4, and PIF5 are also negative phytochrome-interacting protein and can negatively regulates Chl biosynthesis^32^,^33^,^34^. Because phytochrome drives the assemblage of chloroplast and enhancement of Chl content^35^, we infer that BnaC08g49180D and BnaC08g34850D are good candidate genes for the yellowish-leaf trait because they might control the reaction of phytochrome and alter Chl content. DNA sequencing experiments revealed that there is no sequence difference for BnaC08g49180D and one amino acid residual difference at the site +97 for BnaC08g34850D between the *cde1* mutant and ‘ZS11’, thus more focus need to be put on BnaC08g34850D in future to explore its relationship with the mutation of *cde1*.

In the A09 segment of *B. napus* cv. ‘Darmor-*bzh*’, BnaA09g42360D may be related to Chl biosynthesis because it is homologous to AT1G60550, which encodes DHNS (DHNA synthase) that is involved in phylloquinone biosynthesis and research has confirmed that it participated in Chl biosynthesis^30^. As BnaA09g42360D has no homologous genes in the other four homologous segments, it was not deemed a candidate gene of the *CDE1* locus in the present study.

Four genes (BnaC08g34760D, BnaC08g34770D, BnaC08g34780D, and BnaC08g49190D) in the *CDE1* mapping interval of the C08 segment of *B. napus* cv. ‘Darmor-*bzh*’ encode proteins of unknown function, which therefore could not be excluded because their relationship with the yellowish-leaf mutant is unknown to date. Furthermore, other genes with functionalities such as transcription factor or RNA splicing, etc, in the mapping interval, cannot also be excluded from candidate gene list before the gene responsible for the yellowish leaf phenotype is demonstrated.

## Discussion

Seedlings of the *cde1* mutant isolated from an EMS-mutagenized line exhibit a yellowish-leaf phenotype and Chl deficiency. At the budding stage, *cde1* leaves gradually turn green. The leaf colour trait of *cde1* is controlled by one dominant gene, rather than recessive genes, such as *Cr*, *BnChd1*, and *BnaC.ygl*^26^,^27^,^28^ in *Brassica napus*. To date, research has showed that most leaf colour mutations are caused by recessive mutated genes, whereas few are controlled by dominant genes. Because leaf colour cannot be restored to green in a heterozygote, it might be concluded that the locus controlling leaf colour does not consist of genes involved in biosynthesis from glutamyl-tRNA to Chls a and b^6^. In our work, BnaC08g34850D which belongs to bHLH transcription factor may be regarded as candidates for dominant *CDE1* locus. Previous reports revealed that PIFs as members of bHLH transcription factor family act as negative regulators of Chl biosynthesis by directly binding to G-box motifs in the *PORC* (protochlorophyllide oxidoreductase) and *FeChII* (ferrochelatase) promoters in Chl biosynthetic pathway, may lead to yellowish leaf phenotype^36^. Our former iTRAQ-based quantitative proteomics analysis of the *cde1* mutant and its corresponding wild-type has demonstrated that the gene expressions in Chl biosynthesis and photosynthesis pathways^20^ were down-regulated in the yellowish leaves. These results are consistent with theoretical prediction analysis about the candidates. Thus BnaC08g34850D in the mapping interval may be probably the important candidate gene for the dominant yellowish locus. However, the unknown protein genes and other genes functioning as transcription factor or RNA splicing, etc, within the mapping interval, cannot be excluded.

The use of SNP markers has benefited plant genotyping efforts because of the numerous distinct markers and high genome coverage^37^,^38^,^39^,^40^,^41^. Whether obtained from chip hybridization or DNA sequencing experiments, SNP marker data have been commonly used for gene or locus identification and molecular breeding. The Brassica 60 K SNP BeadChip Array has helped to advance rapeseed research efforts^42^,^43^,^44^. It has recently enabled the efficient construction of several high-quality saturated linkage maps over a short period^45^,^46^,^47^,^48^,^49^. Our current study also produced a saturated *B. napus* map, with 2,878 bins containing 13,347 SNP markers and a total length of 1,968.6 cM. We used 89 rapeseed plants from a segregating population for SNP genotyping and linkage mapping. We then developed SSR markers for fine-mapping of the target locus. We pre-mapped the locus using SNP markers and fine-mapped the locus with SSR markers based on the genome sequence. We adopted this mapping strategy by linking SSR markers with SNP markers. This represents a good mapping strategy because of the associated reduced SNP genotyping costs and convenience of using SSR or other markers. Additionally, designed SNP polymorphic markers may have low coverage in the mapping interval.

Our findings provided important information regarding the gene responsible for the yellowish-leaf mutation based on the mapped interval and genetic information. However, the mapped interval length of 311 kb is likely too long to enable the identification of specific genes for subsequent gene function evaluations. In fact, because of the lack of polymorphic SNP or SSR markers, the mapping interval obtained is probably the shortest possible interval for the mapping population used. To further shorten the mapped interval, one strategy could be to use parents with polymorphic markers to construct a new inheritance population for mapping. Another strategy could involve resequencing of the mutant accession to confirm the sequence differences that regulate the phenotypic variation in leaf colour.

*B. napus* originated approximately 7,500 years ago by hybridization between *B. rapa* and *B. oleracea*, followed by chromosome doubling^50^. *B. rapa* and *B. oleracea*, as parents of *B. napus*, are highly related evolutionarily. Based on this knowledge, it can be concluded that short segments, such as that listed herein (less than 312 kb), are usually conserved structurally among species and contain a parallel set of genes. Therefore, comparisons among closely related homologous segments are beneficial in that they allow replenishment of annotation information for species with incomplete information in the genome database. The comparisons we conducted generated gene annotation information and allowed gap filling. As such, our results on the gene annotation based on the alignments are reliable and necessary for finding the candidate genes. In our work, we found that the homologous segments on A09 and C08 chromosomes contain nearly the same set of genes, but the homologous segments on A09 chromosomes contain smaller gene sets than those on the C08 segments. This was likely the result of genome (A and C) evolution in the *Brassica* genus.

## Methods

### Plant materials

The *B. napus* Chl-deficient mutant, *cde1*, was originally isolated from EMS-mutagenized *B. napus* line NJ7982 at Nanjing Agricultural University in China. The yellowish-leaf *cde1* mutant was crossed with the canola variety ‘ZS11’, then selfed to produce the F_2_ generation. We phenotyped the F_2_ population plants at the 6-week-old stage, and DNA from 13 wild-type plants and 76 mutants, and DNA from the recessive parent ‘ZS11’, was selected for SNP genotyping. In total, 38 wild-type and 26 mutant plants of the F_2_ population at the 20-week-old stage were prepared for Chl content determination. The F_2_ population and its four F_2:3_ family populations were used for genetic segregation analysis. We randomly selected 30 *cde1* mutant plants and 30 wild-type plants from the F_2:3_ populations for analysis of agronomic traits. The 90 individuals for SNP genotyping and 620 individuals from the F_2:3_ families were scanned for SSR markers. All materials were grown in the fields of the Jiangpu Experimental Station at Nanjing Agricultural University. Plants were sown uniformly in a row of 2.5 m length with 15 individuals in each row and the rows were spaced at a distance of 0.4 m.

### Chl content determination

Chl was extracted from 0.2-g samples of fresh leaves using 50 mL of 80% acetone (LingFeng, Shanghai, China). Absorbances of total Chl, Chl a, and Chl b were determined using an established procedure^20^.

### Construction of a SNP map

Total DNA was extracted from fresh leaves using a modified cetyl-trimethylammonium bromide (CTAB) method^51^. The DNA samples were diluted to 200 ng uL^−1^ and then genotyped using the Brassica 60 K SNP BeadChip Array. The DNA sample preparation, hybridization to the BeadChip, and imaging of the arrays were completed by the Beijing Emei Tongde Development Co. Ltd. (Beijing, China). Allele-calling for each locus was performed using the GenomeStudio genotyping software, v2011.1 (Illumina, Inc.). Cluster definitions were based on genotype data from the rapeseed individuals. The SNP markers were named using “M” plus the index numbers assigned by GenomeStudio.

The polymorphic SNP markers were first sorted into different bins. The first marker within each bin was selected as the representative of the bin and used to construct the LG with JoinMap 4 software^52^. The SNP markers were first grouped at the linkage logarithm of odds 13.5. The marker order and distances in each LG were calculated using JoinMap 4, with the mapping function of Kosambi and the Regression mapping algorithm.

### Mapping of the *CDE1* locus

Based on the SNP map, the *CDE1* locus was determined and potential physical regions containing the *CDE1* locus were identified by aligning SNP probe sequences and co-segregated SNP markers in the genomes of *B. napus* and *B. oleracea* (one ancestral parent of *B. napus*). The interval sequence was downloaded (http://www.genoscope.cns.fr/brassicanapus/data/ and http://brassicadb.org/brad/downloadOverview.php) for bioinformatics analysis and SSR primer development to confirm the interval detected for *CDE1*.

Based on the sequences of the physical regions of *B. napus* containing the *CDE1* locus, 1,621 SSR loci were identified using SSRHunter 1.3 software^53^ with a 6-bp motif maximum and three repeat minimum. A total of 135 SSR loci with a 150 bp sequence on both sides were selected to design primers using the Primer Premier 5.0 software^54^. The PCR conditions were as follows: denaturation at 95 °C for 10 min, followed by 35 cycles of 95 °C for 30 s, annealing for 40 s, and 72 °C for 40 s and a final extension step at 72 °C for 10 min. Two SSR markers, BnC08Y56 (223 bp expected size; forward primer: 5′-TTTAACCGGGACTTGAGA-3′; reverse primer: 5′-TTGGGCTAATGAACCTTT-3′; 49.8 °C annealing temperature) and BnC08Y66 (249 bp expected size; forward primer: 5′-GAGGAGCGACAAGATGAA-3′; reverse primer: 5′-TAAGTACCACCGAAAGCA-3′; 49.2 °C annealing temperature), were polymorphic and used to genotype the population and fine-map the *CDE1* locus. The recombinant individuals identified by SSR marker scanning were further genotyped by the Brassica 60 K SNP BeadChip Array.

### *Brassica* segments homologous to the mapped interval and candidate gene analysis

Whole genome sequences of *B. rapa*, *B. oleracea, B. napus* cv. ‘Darmor-*bzh*’, and *B. napus* cv. ‘ZS11’ were downloaded from a public database (http://brassicadb.org/brad/, http://www.genoscope.cns.fr/brassicanapus/, and http://www.ncbi.nlm.nih.gov/assembly/GCA_000686985.1)^50^,^55^,^56^. The alignment of sequences containing the *CDE1* locus to genomic sequences was performed using BLASTN (http://blast.ncbi.nlm.nih.gov/). Segments covering all adjoining similar sequences with an E value ≤ 1e-50 were considered homologous segments in the corresponding genome. The similarity of these homologous segments was assessed by dot matrix analysis. A bioinformatics analysis of annotated genes in these homologous regions was then completed.

## Additional Information

**How to cite this article**: Wang, Y. *et al*. Fine mapping of a dominant gene conferring chlorophyll-deficiency in *Brassica napus*. *Sci. Rep*. **6**, 31419; doi: 10.1038/srep31419 (2016).

## Supplementary Material

Supplementary Information

Supplementary Table S1

## Figures and Tables

**Table 1:** Chl content in the leaves of *cde1* mutants and wild-types in the F_2_ population in *Brassica napus*.

Genotype	Chl a (mg/g)	Chl b (mg/g)	Total Chl (mg/g)	Chl a/b ratio
*cde1*	0.92 ± 0.20	0.20 ± 0.04	1.12 ± 0.24	4.66 ± 0.52
wild-type	1.14 ± 0.18**	0.23 ± 0.04**	1.36 ± 0.21**	5.08 ± 0.53**

**Table 2:** Difference in agronomic traits between the yellow and green-leaf seedlings in the F_2:3_ populations of *Brassica napus*.

Trait	*cde1*	wild-type
plant height (cm)	152.09 ± 16.74	158.57 ± 15.11
height of effective branch (cm)	49.51 ± 13.57	51.87 ± 30.59
first branch number	6.93 ± 1.55	6.93 ± 1.48
siliques of main inflorescence	56.10 ± 14.62	60.27 ± 14.73
total siliques per plant	345.67 ± 141.05	405.67 ± 141.97
1,000-seed weight (g)	3.81 ± 0.47	4.07 ± 0.46*
yield per plant (g)	18.16 ± 10.72	26.48 ± 17.59*

‘*’ indicates a significant difference between the two groups at the 0.05 probability level by *t*-test. (mean ± SD, *n* = 30 for each sample).

**Table 3:** Segregation of the F_2_ generation and its four F_2:3_ families of *Brassica napus*.

Population	F_2_	F_2:3_ (1)	F_2:3_ (2)	F_2:3_ (3)	F_2:3_ (4)
Number of yellowish-leaf plants	119	419	144	42	337
Number of green-leaf plants	38	155	58	15	133
Total number	157	574	202	57	470
Expected segregation ratio	3:1	3:1	3:1	3:1	3:1
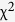	0.02	1.12	1.29	0.01	2.55
*P* value	0.89	0.29	0.26	0.94	0.11

**Table 4:** Basic information for 19 linkage groups constructed with the (‘ZS11’ × *cde1*) F_2_ population of *Brassica napus*.

LG	Bin	Marker	Length (cM)	Mean interval (cM)	Max interval (cM)	Min interval (cM)
A01	130	542	127.0	0.962	32.277	0.046
A02	136	422	111.6	0.826	8.635	0.004
A03	295	957	165.8	0.564	7.685	0.003
A04	223	759	86.7	0.390	4.672	0.010
A05	180	588	92.2	0.515	4.182	0.004
A06	240	754	125.0	0.523	8.167	0.006
A07	218	602	107.4	0.495	6.935	0.021
A08	131	721	100.8	0.775	8.216	0.004
A09	290	1098	121.5	0.421	5.653	0.010
A10	169	1004	108.9	0.648	11.850	0.011
C01	71	428	92.0	1.314	14.030	0.087
C02	34	218	41.8	1.268	15.321	0.136
C03	190	1184	173.9	0.920	7.229	0.040
C04	102	695	97.7	0.967	9.006	0.064
C05	107	399	124.5	1.174	5.438	0.061
C06	78	434	49.7	0.645	3.963	0.009
C07	136	1252	112.8	0.835	11.073	0.083
C08	97	831	83.7	0.872	5.287	0.024
C09	51	459	45.8	0.916	3.656	0.077
Total	2,878	13,347	1,968.6			

**Table 5:** Genes on the mapped segments and their homologous *Brassica* segments.

C08 segment in *B. napus* cv. ‘ZS11’	C08 segment in *B. napus* cv. ‘Darmor-*bzh*’	C08 segment in *B.oleracea*	A09 segment in *B. napus* cv. ‘Darmor-*bzh*’	A09 segment in *B.rapa*	*Arabidopsis thaliana* homologue	Encode	Putative molecular function
existence 30668117–30669561	BnaC08g34740D 32779893–32781337	Bol045819 34949314–34949730	BnaA09g42280D 29404630–29405046	Bra039937 33238896–33239312	AT2G22880	VQ12	response to UV-B
existence 30676433–30671875	BnaC08g34750D 32783666–32788354	existence 34957739–34953181	BnaA09g42290D 29406635–29411965	Bra039936 33240924–33245982	AT3G10450	SCPL7, SERINE CARBOXYPEPTIDASE-LIKE 7	proteolysis, serine-type carboxypeptidase activity
existence 30683953–30684165	BnaC08g34760D 32795838–32796050	existence 34965259–34965471					
existence 30687032–30689768	BnaC08g34770D 32798919–32801655	Bol045820 34968577–34970820	BnaA09g42300D 29414646–29417671	Bra039935 33251744–33254092	AT2G22795	unknown protein	
existence 30691711–30691066	BnaC08g34780D 32802950–32803846	Bol044727 34972118–34973014	BnaA09g42310D 29418582–29419775	Bra039934 33255506–33256402	AT2G22790	unknown protein	
				Bra039933 33258113–33259127	AT5G15690		zinc ion binding
existence 30699472–30700359	BnaC08g34790D 32808815–32810013	Bol045821 34977983–34978885	BnaA09g42320D 29426463–29427966	Bra039932 33264857–33265789	AT3G43590	zinc knuckle (CCHC-type) family protein	nucleic acid binding, zinc ion binding
existence 30707301–30705253	BnaC08g34800D 32814704–32816754	Bol044728 34983880–34985921	BnaA09g42330D 29428392–29430445	Bra039931 33266424–33268478	AT1G78490	CYP708A3,CYTOCHROME P450, FAMILY 708, SUBFAMILY A, POLYPEPTIDE 3	oxygen binding, heme binding, iron ion binding, monooxygenase activity, oxidoreductase activity, acting on paired donors, with incorporation or reduction of molecular oxygen
existence 30718661–30718329	BnaC08g34810D 32827771–32828105	Bol044729 34997133–34998224			AT3G07860	Ubiquitin-like superfamily protein	RNA splicing, mRNA processing
existence 30719610–30719097	BnaC08g34820D 32828163–32829449	existence 34998224–34997712			AT3G07860	Ubiquitin-like superfamily protein	RNA splicing, mRNA processing
	BnaC08g34830D 32829688–32831487	existence 34998856–35000000			AT2G32295	EXS (ERD1/XPR1/ SYG1) family protein	unknown function having several predicted transmembrane domains and is predicted to be mitochondrial
existence 30730524–30732419	BnaC08g34840D 32839051–32840950	existence 35008220–35010119	BnaA09g42340D 29445156–29447306	Bra039930 33294976–33296852	AT5G42580	CYP705A12,CYTOCHROME P450, FAMILY 705, SUBFAMILY A, POLYPEPTIDE 12	oxygen binding, heme binding, iron ion binding, oxidoreductase activity, acting on paired donors, with incorporation or reduction of molecular oxygen, NAD(P)H as one donor, and incorporation of one atom of oxygen
existence 30763743–30777223	BnaC08g49140D C08_random 4028479–033220	Bol045822 35029708–35034451	BnaA09g42350D 29458763–29463263	Bra039929 33310195–33314710	AT1G78500	PEN6, PENTACYCLIC TRITERPENE SYNTHASE 6	catalytic activity, lupeol synthase activity, oxidosqualene cyclase activity
existence 30782725–30784035	BnaC08g49150D C08_random 4038699–4040009	Bol045823 35039932–35041242		Bra039928 33319068–33320375	AT3G30280	HXXXD-type acyl-transferase family protein	transferase activity, transferring acyl groups other than amino-acyl groups
existence 30805943–30804351	BnaC08g49160D C08_random 4065984–4067799	Bol044730 35072123–35073711	existence 29470643–4065985	Bra039927 33324120–33325712	AT2G22770	NAI1	DNA binding, protein dimerization activity, sequence-specific DNA binding transcription factor activity
			BnaA09g42360D 29487843–29488100		AT1G60550	DHNS, ECHID, ENOYL-COA HYDRATASE/ISOMERASE D	1,4-dihydroxy-2-naphthoyl-CoA synthase activity, isomerase activity
existence 30846881–30846362	BnaC08g49170D C08_random 4084561–4085051	Bol044731 35100776–35101228			AT5G20980	ATMS3, METHIONINE SYNTHASE 3, MS3	5-methyltetrahydropteroyltriglutamate-homocysteine S-methyltransferase activity, methionine synthase activity, zinc ion binding
existence 30854671–30853301	BnaC08g49180D C08_random 4092893–4094259	Bol044732 35107886–35109256	BnaA09g42370D 29504480–29505847	Bra039926 33345880–33347251	AT2G22760	basic helix-loop-helix (bHLH) DNA-binding superfamily protein	DNA binding, protein dimerization activity, sequence-specific DNA binding transcription factor activity
existence 30887147–30881446	BnaC08g49190D C08_random 4097277–4101753	Bol044733 35112570–35117392	BnaA09g42380D 29509356–29514450	Bra039925 33349873–33354665	AT5G43745	Protein of unknown function (DUF1012)	
existence 30898460–30907207	BnaC08g34850D32919418–32920971	Bol044734 35134202–35135529	BnaA09g42390D 29524526–29525841	Bra039924 33362502–33364043	AT2G22750	basic helix-loop-helix (bHLH) DNA-binding superfamily protein	DNA binding, protein dimerization activity, sequence-specific DNA binding transcription factor activity
existence 30930933–30928962	BnaC08g34860D 32933021–32936306	Bol044735 35147908–35152898	BnaA09g42400D 29534356–29537737	Bra039923 33375941–33378194	AT2G22720	SPT2 chromatin protein	
existence 30933664–30933049	BnaC08g34870D 32937075–32938479	existence 35152898–35152024			AT5G06480	Immunoglobulin E-set superfamily protein	Lipid recognition
existence 30935111–30934732	existence 32939489–32939108	Bol044736 35153896–35154277	BnaA09g42410D 29538544–29538988	Bra039922 33381024–33381265	AT2G22640	ATBRK1, BRICK1, BRK1, HSPC300	identical protein binding, protein binding
existence 30943739–30940048	existence 32952848–32947998	existence 35168163–35162564	existence 32952848–32947998	Bra039921 33384381-33389807	AT2G22630	AGAMOUS-LIKE 17, AGL17	DNA binding, protein dimerization activity, sequence-specific DNA binding transcription factor activity
existence 30964212–30973897	BnaC08g34880D 32967612–32974266	Bol045824 35182743–35188397	BnaA09g42420D 29565850–29569644	Bra038506 33412450–33418101	AT2G22620	Rhamnogalacturonate lyase family protein	carbohydrate binding, lyase activity

‘existence’ indicates a gene homologous to the same table row, existed but was un-annotated on the location. The six genes of the homologous segment in the unanchored “chrC08_random” pseudo-molecule were filled in the gap of the C08 segment in *B. napus* cv. ‘Darmor-*bzh*’ by the functional comparison with their homologous genes in other homologous segments.
