# Supplementary material for: Fine mapping of a dominant gene conferring chlorophyll-deficiency in Brassica napus
Source: Sci Rep. 2016 Aug 10;6:31419. doi: 10.1038/srep31419 (PMC4979034; doi:10.1038/srep31419)
Supplement: Supplementary Information [file srep31419-s1.pdf]

Fine mapping of a dominant gene conferring chlorophyll-deficiency in *Brassica napus*

Yankun Wang[1], Yongjun He[1], Mao Yang[1], Jianbo He[2], Pan Xu[1], Mingquan Shao[1], Pu Chu[1] & Rongzhan Guan[1*]

[1]State Key Laboratory of Crop Genetics and Germplasm Enhancement, Nanjing Agricultural University, Nanjing 210095, China; Jiangsu Collaborative Innovation Center for Modern Crop Production, Nanjing, Jiangsu, China,
[2]Soybean Research Institute, Nanjing Agricultural University, Nanjing 210095, Jiangsu, China.

[*]Correspondence should be addressed to Rongzhan Guan (e-mail: guanrzh@njau.edu.cn)

Supplementary Figure S1 Dot matrix of the homologous segments

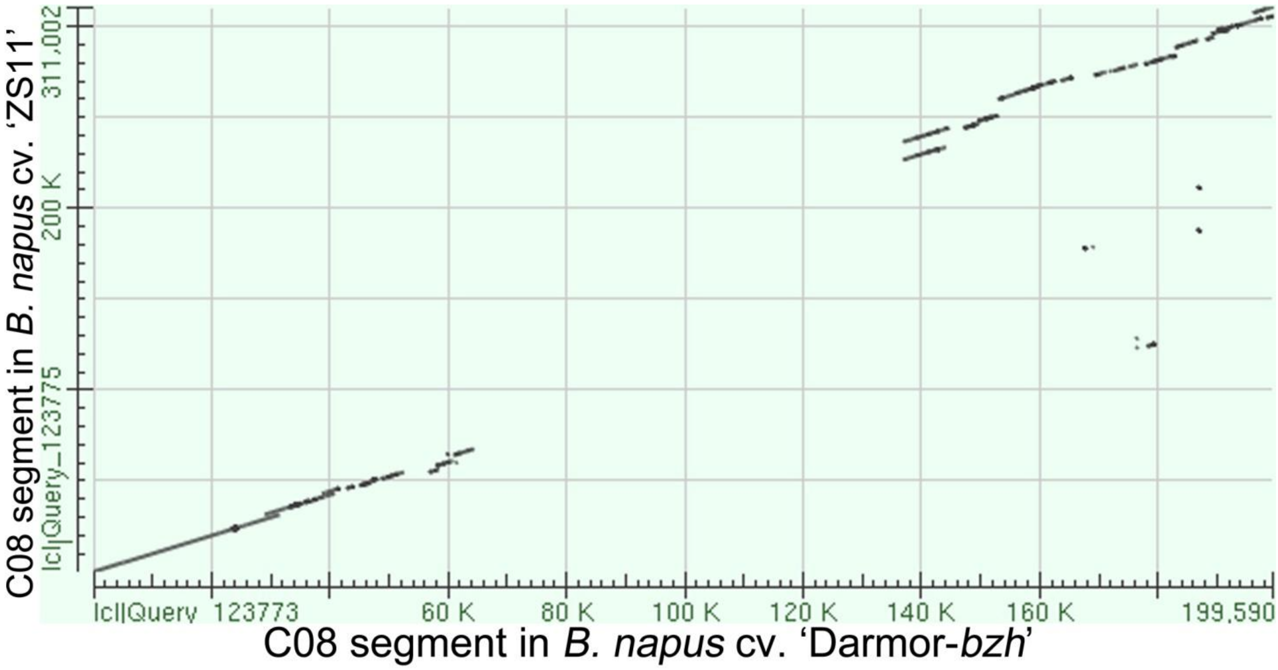

a

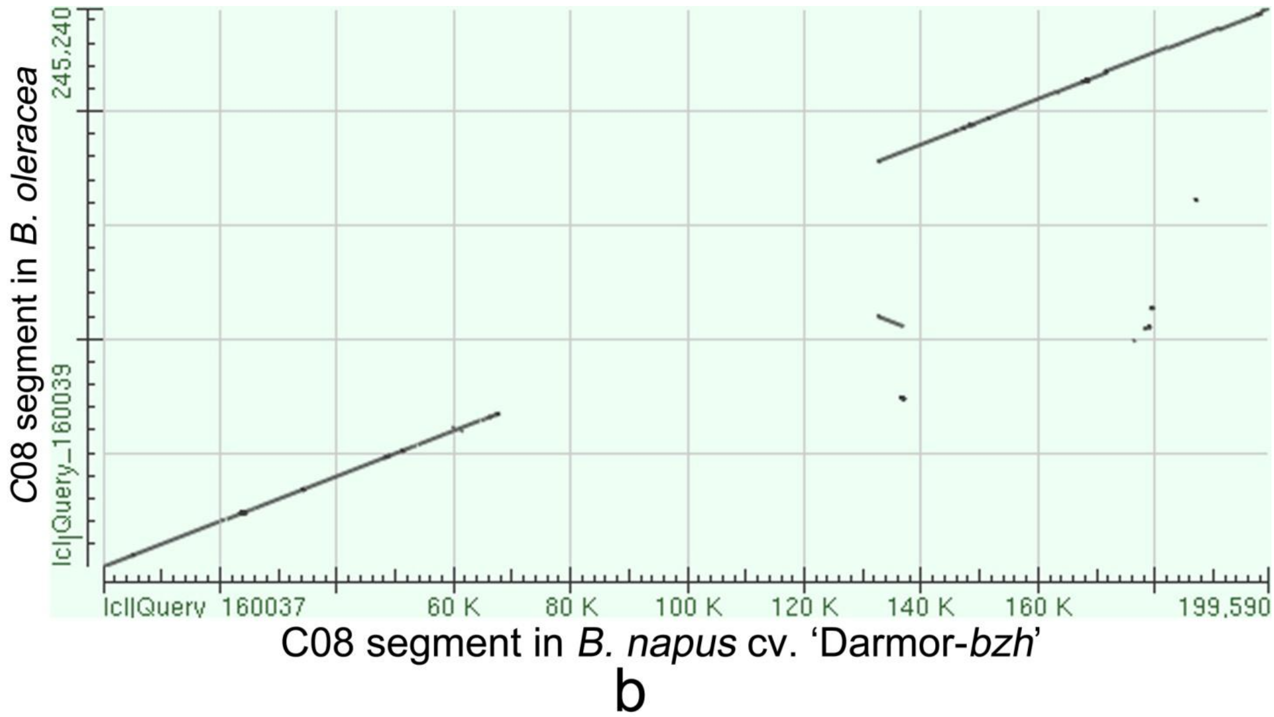

b

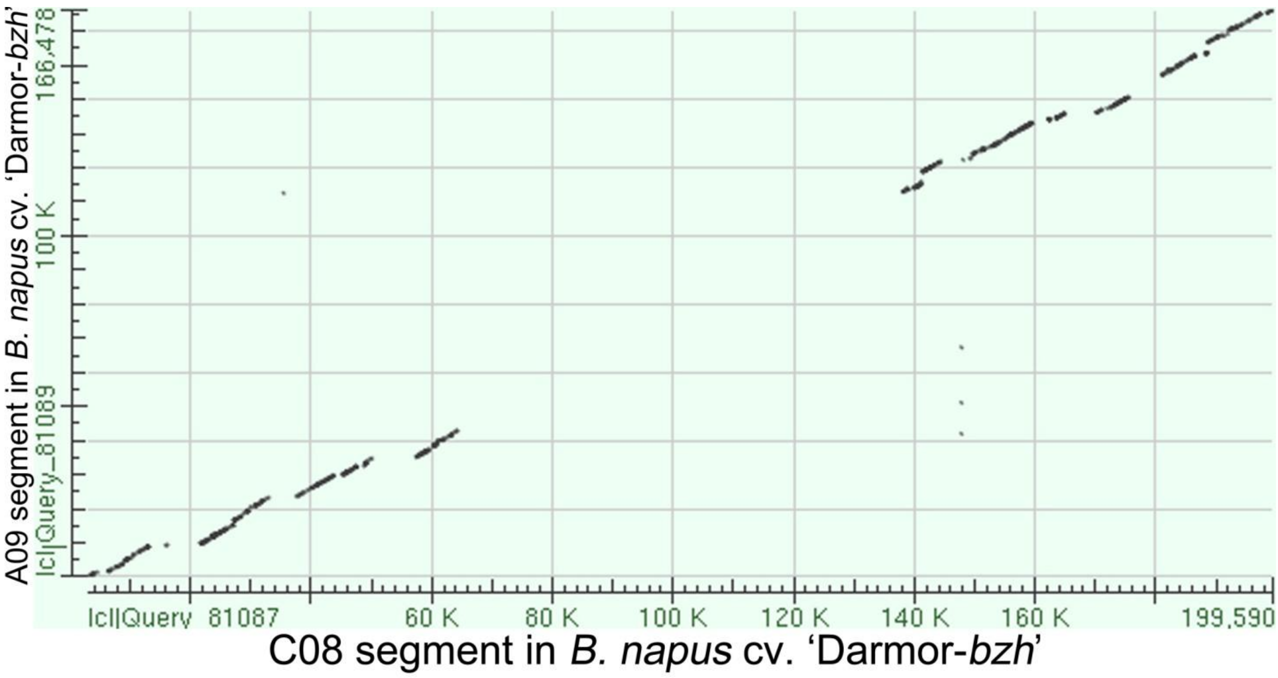
A09 segment in B. napus cv. 'Darmor-bzh'
lcl|Query_81089
166,478
100 K
lcl|Query 81087
60 K
80 K
100 K
120 K
140 K
160 K
199,590
C08 segment in B. napus cv. 'Darmor-bzh'

c

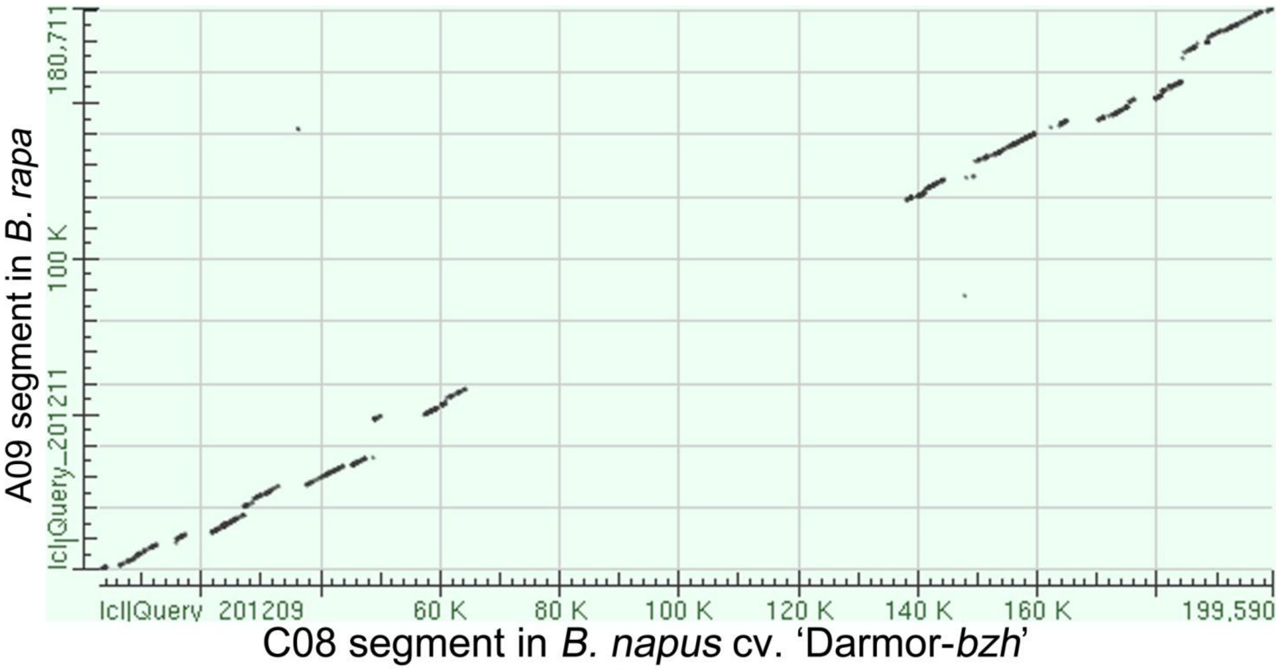
A09 segment in B. rapa
lcl|Query_201211
180,711
100 K
lcl|Query 201209
60 K
80 K
100 K
120 K
140 K
160 K
199,590
C08 segment in B. napus cv. 'Darmor-bzh'

d

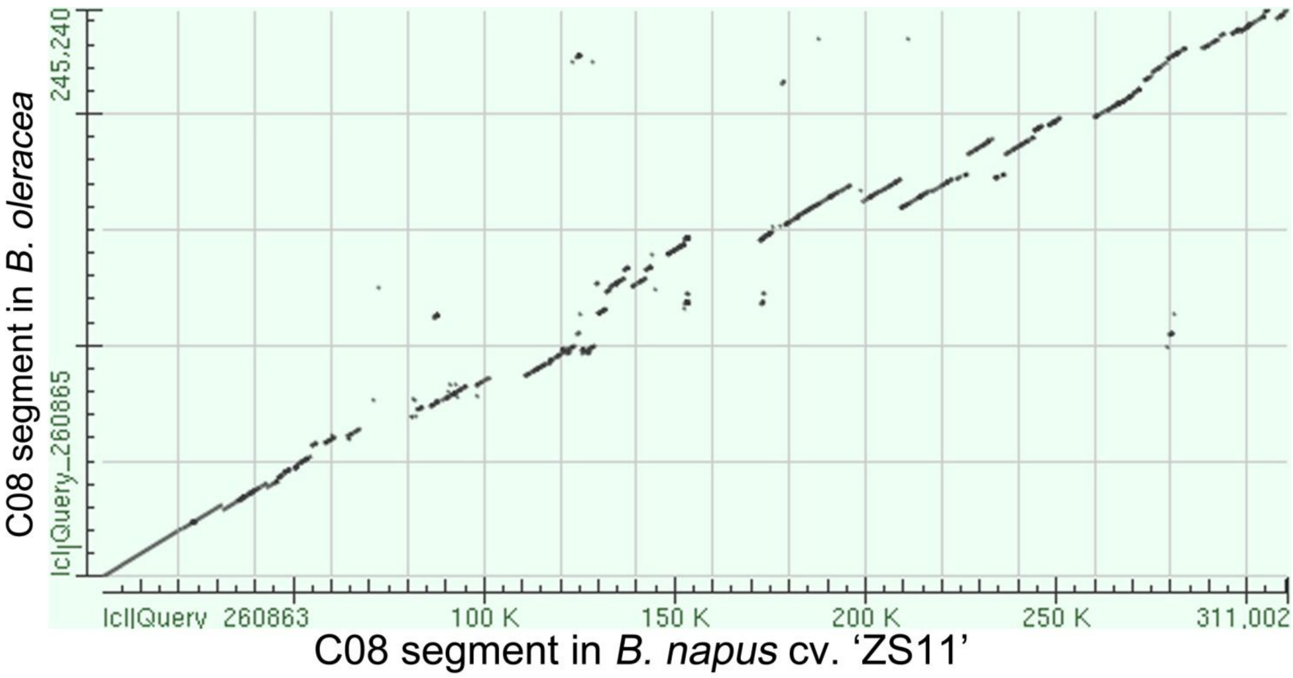
C08 segment in B. oleracea
245,240
Icl|Query_260865
Icl|Query 260863
100 K
150 K
200 K
250 K
311,002
C08 segment in B. napus cv. 'ZS11'

e

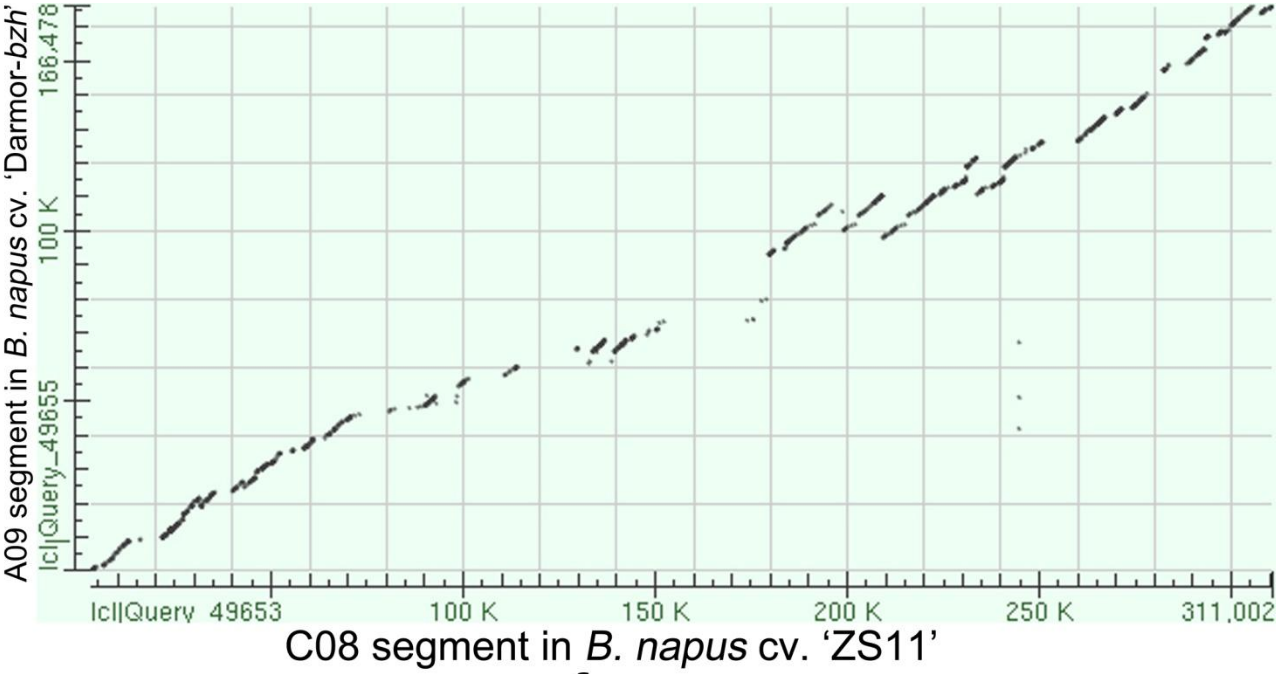
A09 segment in B. napus cv. 'Darmor-bzh'
166,478
100 K
Icl|Query_49655
Icl|Query 49653
100 K
150 K
200 K
250 K
311,002
C08 segment in B. napus cv. 'ZS11'

f

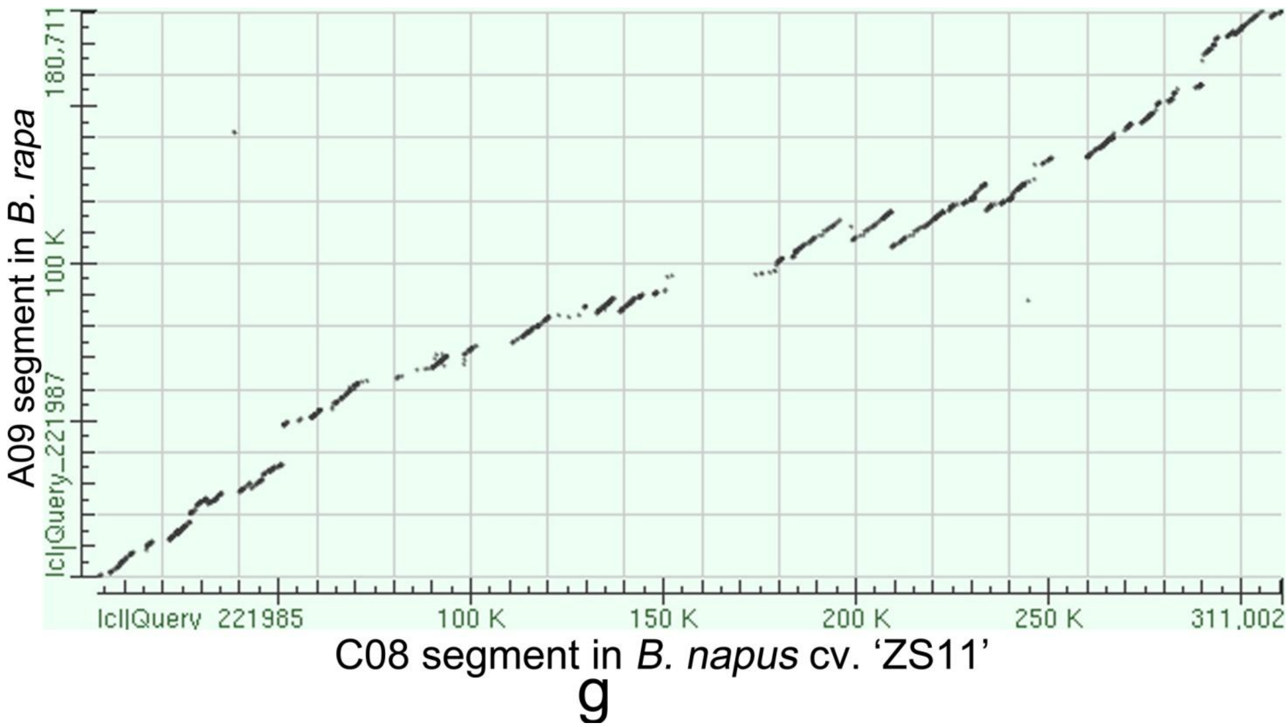
180,711
100 K
100
Icl|Query_221987
A09 segment in B. rapa
Icl|Query 221985
100 K
150 K
200 K
250 K
311,002
C08 segment in B. napus cv. 'ZS11'

g

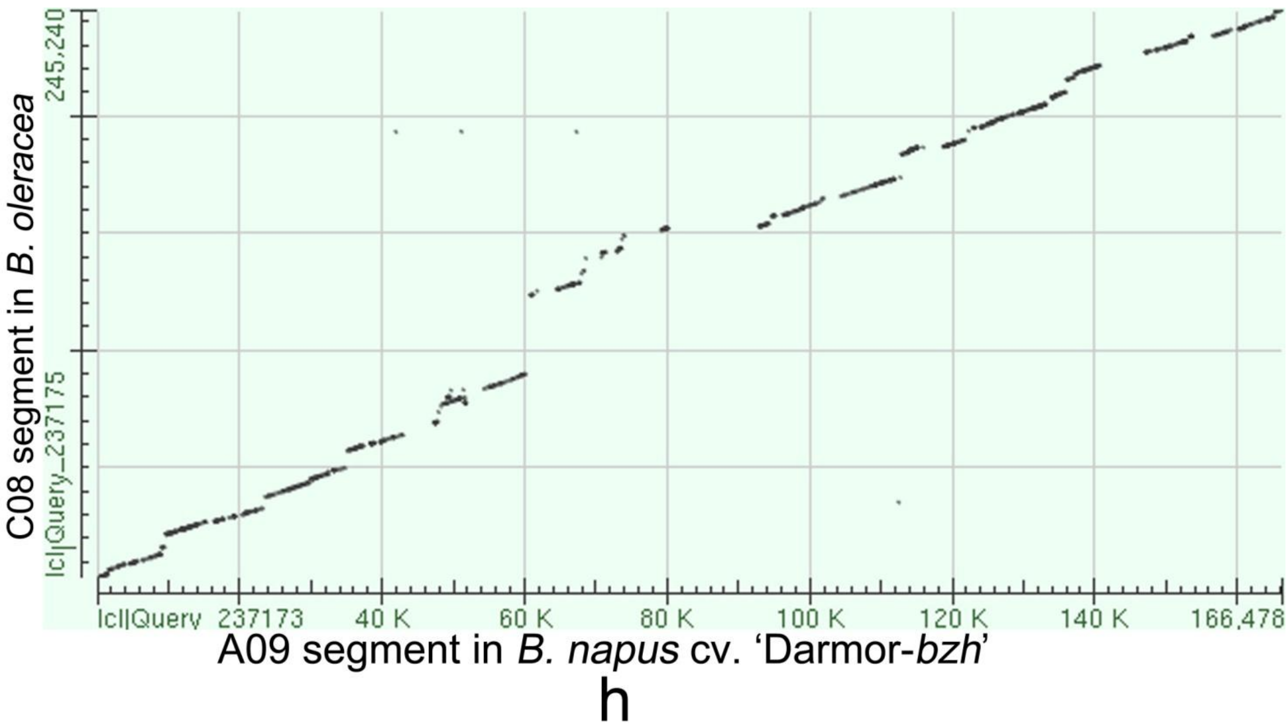
245,240
Icl|Query_237175
C08 segment in B. oleracea
Icl|Query 237173
40 K
60 K
80 K
100 K
120 K
140 K
166,478
A09 segment in B. napus cv. 'Darmor-bzh'

h

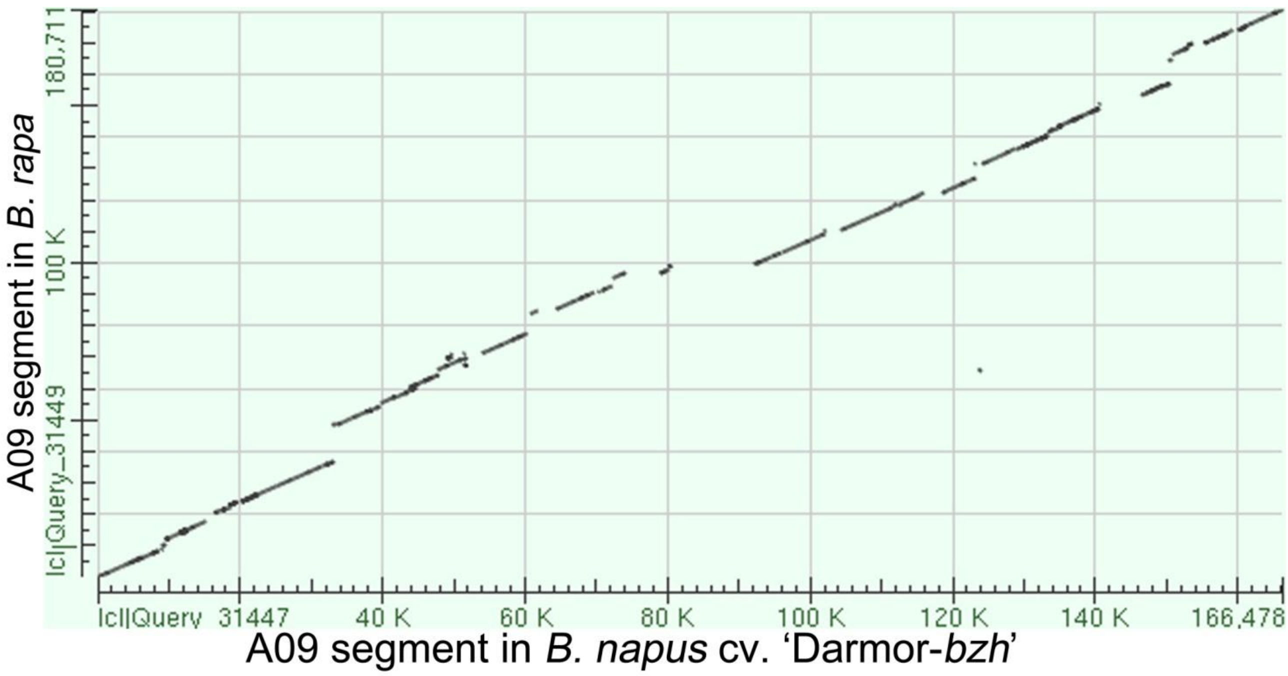
A09 segment in B. rapa
180,711
100 K
100
Icl|Query_31449
Icl|Query 31447
40 K
60 K
80 K
100 K
120 K
140 K
166,478
A09 segment in B. napus cv. ‘Darmor-bzh’

i

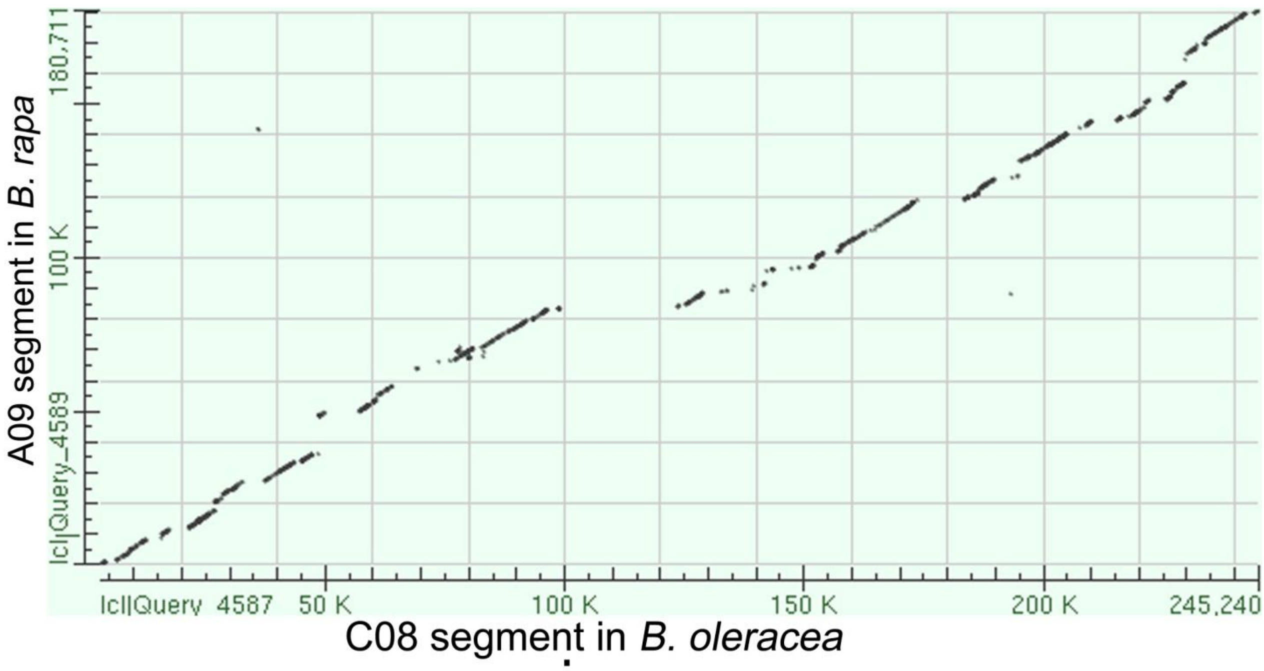
A09 segment in B. rapa
180,711
100 K
100
Icl|Query_4589
Icl|Query 4587
50 K
100 K
150 K
200 K
245,240
C08 segment in B. oleracea

j

Supplementary Figure S2 Dot matrix of segment in C08_random pseudo-molecule to other homologous segments

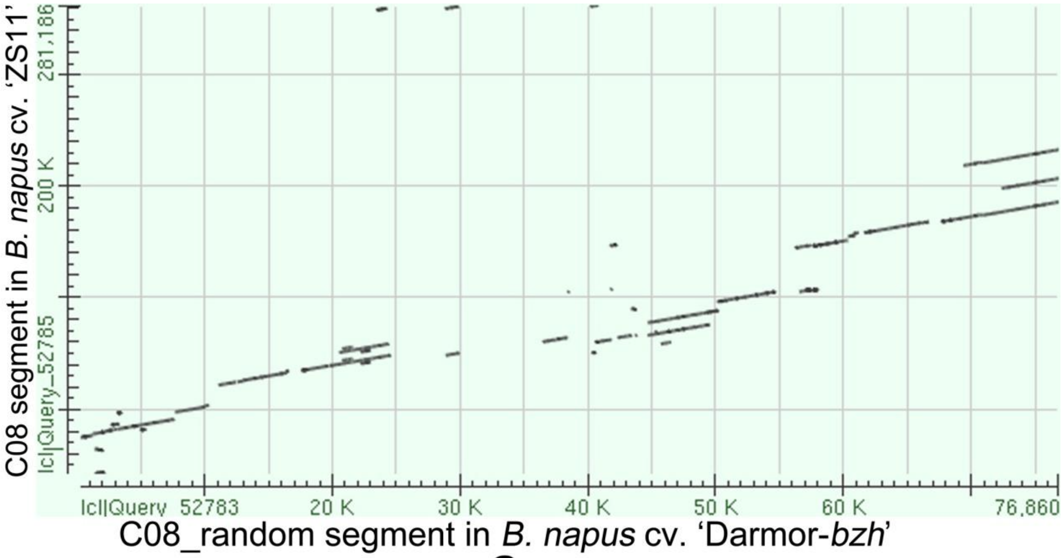

a

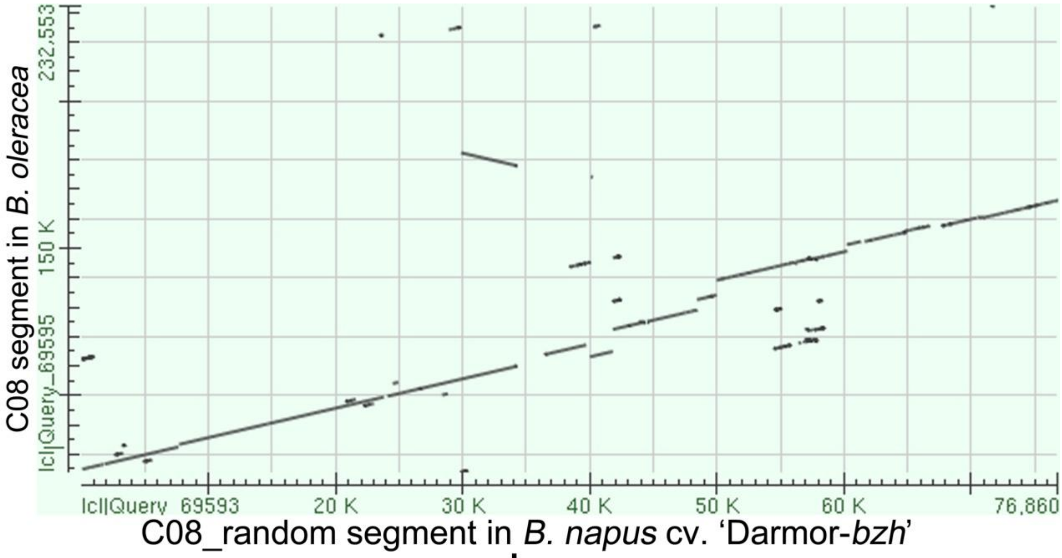

b

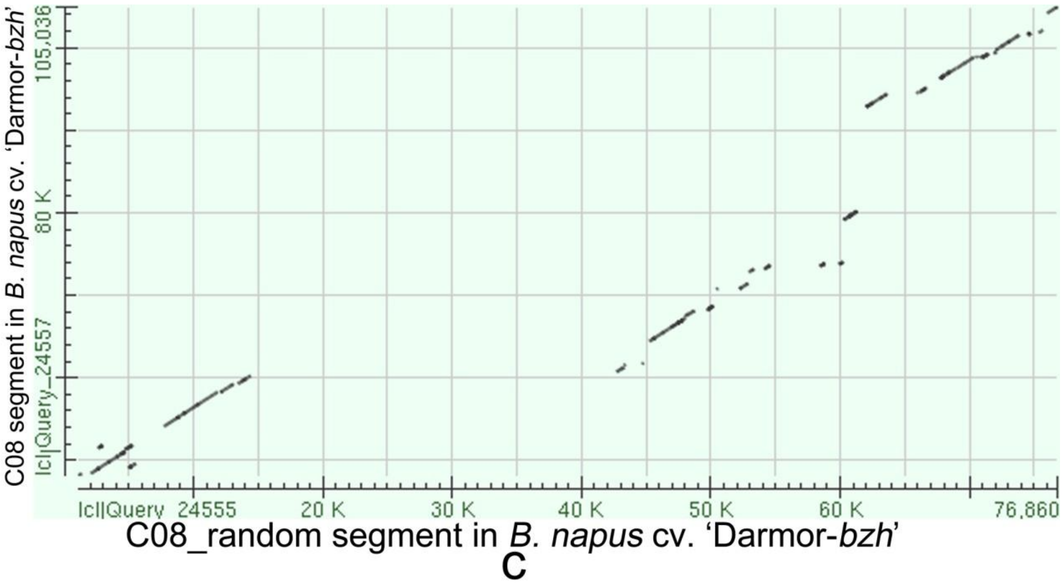

c

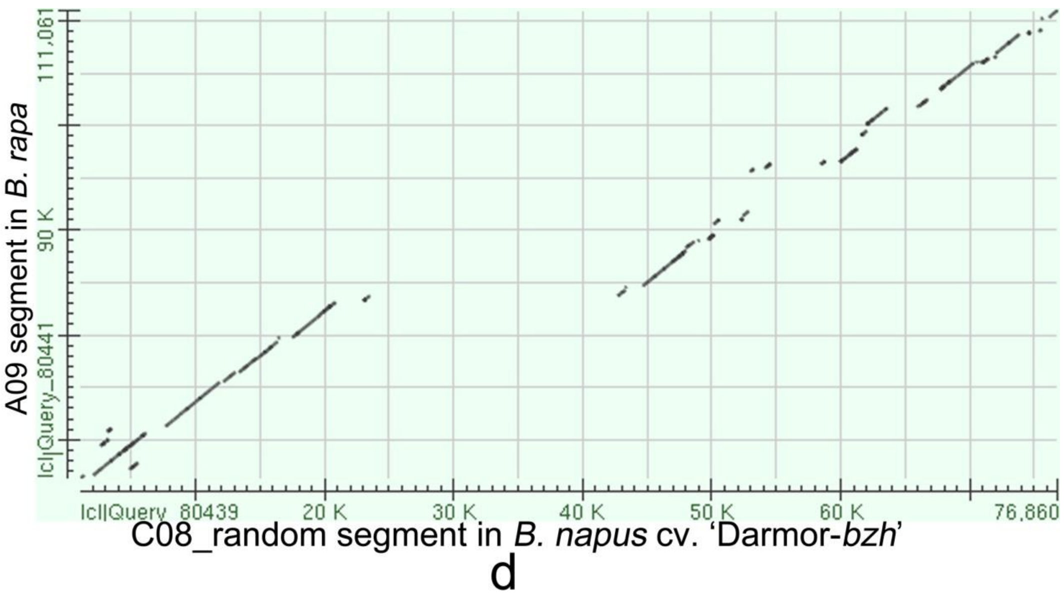
A09 segment in *B. rapa*
lcl|Query_80441
90 K
111,061
lcl|Query_80439
20 K
30 K
40 K
50 K
60 K
76,860
C08_random segment in *B. napus* cv. 'Darmor-*bzh*'

d

Supplementary Table S1. Information of designed SSR markers

SSR marker	5' primer	3' primer	product size (bp)
BnC08Y8	CGATGTCAATAAAAGTAA	CACTATAACTAATCTAAATGC	250
BnC08Y52	TACTGCCCTTTTCACCAT	GGAAGTTTGACTGCGTTT	234
BnC08Y56	TTTAACCGGGACTTGAGA	TTGGGCTAATGAACCTTT	223
BnC08Y60	TTCCGCATTACACGATAT	GAAACAAGGCTGTGAAGTA	258
BnC08Y66	GAGGAGCGACAAGATGAA	TAAGTACCACCGAAAGCA	249
BnC08Y73	CTCCTAATTGTACGGTGGT	GGAGAACTTGTCGGAAAC	285
BnC08Y82	ACTAAGATGGCGGAAAGA	GAGAAATTGCGAAGGAAA	283
BnC08Y128	TTCTTACACCCGTTCTCC	ACGTGGGCTTTCTAGTTG	212
BnC08Y148	GTGTTACTGTTATTTTCGGATTT	CAGGTGGAGAAGCAAAGG	259
BnC08Y176	GTATCTTTTGGTTCAGCA	TAAATGGTCTTCTTGGTC	224
BnC08Y182	CTTCTTTCCATCCCGCCTCT	ACGTCGCTGGGTTTCTCCA	231
BnC08Y183	CGTTTCTGACGACGAGTTTG	TTGAGTCCTCTATCGGCATCT	171
BnC08Y191	GTAGCAGAAGCGGGGATA	AACGCATTGACCAGCACA	223
BnC08Y197	TGGATATTTTGGCAACTC	ACGCCTAATACTGTTACGAC	164
BnC08Y202	TGGCTGCTTTATTATCCT	TTGCCATCAAAACCTTAT	192
BnC08Y209	GAGTGAACCATTGAAGCAAA	TGGAACCGTTAGGAGACG	240
BnC08Y214	CTGGTAACTGGGCGAGGAG	CCTGCCAATCAGAAATCG	229
BnC08Y219	GACATCCCAATTTCCCTG	ACCCAAGAGTGCTTACGG	247
BnC08Y235	TGTTAGAATAGCTTGTTAGG	ACATTTCACCAAGTTTCA	225
BnC08Y252	AACCAAATAATGCTGACA	TAATAAACAACACGAGGA	242
BnC08Y263	TGTAACTCAAAATCTGTA	GATTTGTAATAAGTTCAG	167
BnC08Y273	AAGGAAAAGCAAGAGGAG	AAGATTCTAAGAGCAAGG	206
BnC08Y294	TGAGTGCACCCTCTATTA	TCACGAGTGTCGAGTTTA	223
BnC08Y300	GTTTGCTTGCTCCATCTC	GGCTCCTCCTTGGGTAAT	177
BnC08Y303	GCAACGACCACGGCTTCT	TTCGGATTCTTCAGGATTTG	193
BnC08Y305	TAAACCCTCTAACCCAACT	GAGACACGAAATCGAAGC	210
BnC08Y341	TACCCGAACGAGTATTGA	ATGTTCATCTCGCGTAAA	228
BnC08Y347	GATGCAAATTCTGAAACT	TAAAAGTCCCACAAACAT	173
BnC08Y357	AATACATAAACCAAACGG	ACTCAAATTCCCAAACGA	242
BnC08Y361	ATTTGATGAGCACCTTTG	AATTCTCGAAAACTGGAT	231
BnC08Y362	ATTTGATGAGCACCTTTG	TTTCACGCGAATTTGTTG	203
BnC08Y366	AAATAACTTGATTCGCTCCT	CAACCTACTGTCGCCTTT	174
BnC08Y381	TCCTTGTTTCTTCTCCCACA	TCTGCACGGACACCTCGT	220
BnC08Y386	GCTTAGGTTTTGAGGTGGAG	TGTGCCGAGGTGTCTGTG	226
BnC08Y388	TTAAGGAAGGGCTACGCT	CTGGCTTGAACTCATTAGGATC	174
BnC08Y392	TTTACCCAATGCAGAAGC	CGTAGTATGAACCAGGAGC	221
BnC08Y393	ACGGAAAGGTTGCAGAGG	AGCACAAGTTTGGCGGTA	274
BnC08Y399	GATACGCCACAGAGGACG	GCCAAACCAGCAGACACC	178
BnC08Y406	GCAAACAAGCCGCCAAAT	GGAGAACGCCGGAAGGAG	167
BnC08Y407	ATTGAAAATACTGAGGGAA	TTTGTAAAGGAATGGAAAG	183
BnC08Y412	CTTGATTATTTATCCGAGAT	TGTTATTGTTTTGGCATG	242
BnC08Y437	ACATTTGAGTTCCCAGTA	ACCTATCAAAACATCTCG	237
BnC08Y445	ACTTCTCCGAGCCACATC	AACGCTTTCTTCACCTTCT	224
BnC08Y451	CGAACTGGAAACACGCTAA	AAGGGAACAGACAGACCAAGACA	293

BnC08Y457	GCCCACCCTAGTTCAAAG	ATAACAACCAGGACCAAGTC	204
BnC08Y460	GAAGATCACAGGAAAAGC	ATCGAATCACATACAACTAG	231
BnC08Y463	TCACGCCGACACCATCAG	ATCAAGCCATAAGTAGTTCATC	263
BnC08Y471	ACAAGCAAAATACTACAT	ACAACAGGAATATCACAA	226
BnC08Y475	ATGGACCACCGAAACCCT	CTTGGTAGATCCAGCCCTTAT	227
BnC08Y481	GATGCTGGCCTCATTTCA	TGCCCATAGTCTTGCTGTT	233
BnC08Y485	TTCCCTAAACCGTTCTTG	CTGCCCTTGCGTCTTCAT	256
BnC08Y488	TCCTGAGCATCGCAAAGA	GTATTCCCCATCCACCAT	165
BnC08Y489	GTTCTTGGAGTGTATGATTG	AGAGGTGTTGGAGTAGCG	253
BnC08Y496	GGAACCAATTTAGCGTAC	CTGGGTCACAGACTCACA	179
BnC08Y499	GCCGTTAATTTGCTTCTA	GGGACTCAGCCACTACAT	284
BnC08Y500	ACAGGGTCTGCAATAGTAGCG	TCGGACGGCATTAAAGGT	183
BnC08Y514	TGCGATTCGATCACCTGT	CGCTTCGGAGATTATGGG	251
BnC08Y517	CAACCTTTCTACGTTTGA	TTTGATTTTCGGTGATTC	210
BnC08Y528	ATTCTTTTGACATCCATA	TGTAACAGCTAACACTTC	177
BnC08Y538	TCAATCCCACGAGAAGAAA	GAGAAAGGCAGAAAGTGTAGAG	171
BnC08Y541	CTCCCAATCCTGCTGTCA	GTTCTCCCGCAAATTCTT	200
BnC08Y547	TGAGGTCCCAGATTTTGT	TCCCTGATTTGAATTGCT	209
BnC08Y549	TCAGGGACGCTTGGAGTT	CCAGTTTGTTAGTTGGGATATTTGT	208
BnC08Y570	ACGCATCAAAGAAGAGGACA	TCTGCAATTCGGTCAACTC	232
BnC08Y585	TGTCCTTGTCGGAAATGC	CTCCTCCTGATGGTAACTATGT	222
BnC08Y588	CGGAGCTAACGAAACAGT	CTCGCTTCTTGAGCAAAT	274
BnC08Y592	CAAAGAACCTCGCCAAAG	CACTCGACCACCAAACCT	231
BnC08Y598	TCAACGGTGGACGAAGGT	TGCGGATCAATGAGAAGGATG	241
BnC08Y603	ATCAAGGGTTACTTCTCCA	TTCGCGTTAAACTTCTTC	240
BnC08Y606	CTTGCTCTAACTCCCGTAAC	AGATTTGCTCGGACTTGC	273
BnC08Y610	CGGATGTTACGAGGAGGA	CATTGGCTACGAATTTACT	233
BnC08Y641	AAAAGTTACCCACCAAGA	AATACACCAACCACCATG	208
BnC08Y644	AGGTTTACGTTGTTGAGA	TTCGCTGATTTAGTTTTC	232
BnC08Y648	GAGGGAGGGGAATCCAGA	TTGCCCAGAGTAGCGTTT	239
BnC08Y659	TGATACATCAATCACTGGGTAC	CTTGCGTTAGCGGAATAG	170
BnC08Y666	GCCAGGAAGAAGAGCAGA	CCAGACCCGAAGATGAAC	210
BnC08Y671	GAAATCAAGCAAGCAAAG	CGTCGAGTCCAAAACCAC	208
BnC08Y686	AAAGTAACCCACCCAGAA	TCAATACCACAACCACCA	210
BnC08Y688	TTCATGGTGGTTGTGGTA	ATTGGTTTTGCCCTATTC	165
BnC08Y701	TTTACCAAGTCAAGCACCAT	TACCGAATTGCAGAACGA	281
BnC08Y705	CCCCAACGGTCACAAAGA	CCAACGAATAAACCATAAATAGAAAC	240
BnC08Y709	GCTCATTTTGTATTTTGCAT	GAAACTCTTTGGACCTTG	258
BnC08Y721	ATTTACCAAATCAAGCAC	TAATATCCGTATGAAGCA	194
BnC08Y751	TACCAAATATGACTCAAAAC	TCTTAGAAGGCTCAGAAG	177
BnC08Y761	CAATTTTGAACGAGTTTGGTA	TTCTTTGCATGTTGTGCC	203
BnC08Y801	TGGAATAAAGAACGCAGTAG	CCGGTATCTCCGACAAGTA	181
BnC08Y807	AGGAATCGGACCTTGGAG	GACCTGCTGTTTGCTTTGT	252
BnC08Y829	TTGATTTGGAACTATTTGAG	GTATTGTATTGCCATTCG	207
BnC08Y847	GTTGATAGCCAATCTACACCT	TTCAGAAGCGAACCTTACT	216
BnC08Y878	GGGTGAAAGGCTTAACTG	AGAAAGGGATGTCGAAGA	220

BnC08Y882	TATCCTCACAACCAAATC	TGTCTGTTACAGCAATCT	254
BnC08Y890	AGCCGAAGTTCCTTTACC	AAATGGAGTTGGCCTGAA	222
BnC08Y907	GTTAGATATGGAGGAGGAGTG	AACGACAGATGCGTTTCA	153
BnC08Y924	CATCAATACACTAACCACCAT	CTCCAAGCACCCATAACT	230
BnC08Y931	ATACAGACCAAATAACATAC	TGTGGAGTAAAAGGTAAA	192
BnC08Y967	CCAAGTCAACTAGGTAAC	ATCACCATTCAAGTTATT	175
BnC08Y983	AATTCATTGTAGGAAGCC	AAAATAGGAAATCAGGAG	233
BnC08Y996	TAAAGACATTCGACCGAG	TGATGAACGACAAGGATT	218
BnC08Y1010	GATCAGGAACCGACGAAA	TCACAAAAGCCCACGAGA	191
BnC08Y1027	ACAAGAGGCAGGCATCAC	CACTTCAAGTTCGGGAGG	209
BnC08Y1037	CGAAACAGACCTTGTGGA	CGCTACGTGGAATGAATA	211
BnC08Y1064	CCGTTTGTACCACTGCCTTAG	TTGGGATTTGCATCTTGAGC	181
BnC08Y1087	AGAGGAGGAGATGTTGTTATG	TTGTGATCGGTTTGAAGA	253
BnC08Y1088	AAGGCGTGGCGTGGAATA	TTCTGACGAAGCGGAGGC	213
BnC08Y1123	TGATTCCCACAAACTCCA	AACCCAATCTGCATCTCC	220
BnC08Y1157	CATGCCATCGTTCTTGTG	ATCGCTTGAGACTACTTCG	224
BnC08Y1158	ATTTCAAACCCTCGTAAC	TCAATAAAGAAGCGTCAG	260
BnC08Y1160	TGTCCAGAAGCAATAAAA	GTAAAGCATCCTAACCGA	215
BnC08Y1170	GCTTGATTACAAATGATAGAGGGAA	CGAGGTGCGGATTGGATG	215
BnC08Y1180	TAGGCAATAAACGACCAG	ATAACGAGGACATTCACA	189
BnC08Y1185	ACTACGAACCATACACCGAT	ATGCTTTCACTTCCCACA	192
BnC08Y1206	ATAACTCAGAACGAAGAA	ATGTACCTAAAGGGAAAT	206
BnC08Y1214	CGGTAAATAAGCATCCCT	TGCCAAACCAACAAAAGC	163
BnC08Y1249	TCTTGAAGCAGGTGAGTTA	GAAGGAGGCACAGAACAT	202
BnC08Y1300	GCCGCTGGCTTCTTCATT	AGGCGTTGTCTCATGTTTCTTCTA	263
BnC08Y1321	GATCCCTTATCCTCCTCATT	CGAACCCACAAACCCATT	208
BnC08Y1360	TCGGTTCGGGTTAAGATC	AGCTCCACCAACTAGGGT	207
BnC08Y1375	AGACCTCTTCTTCGTTTTG	GATCTTCCTCCTGTTCATC	176
BnC08Y1388	ATTCCGTGTCCACAGTCA	ACCTTAAACGAACACCAGA	184
BnC08Y1397	CATATTATTTAAGGGTATTTCCG	CGTTTCTTGCGTAGTTGG	201
BnC08Y1409	TGCTAAAGCTGCTCCTCC	TGAAAGCCACATCCAAGA	161
BnC08Y1416	AAAGTGTCCGCATCCAAG	GAGCCAATCAAATACAAGCA	241
BnC08Y1426	CCGTCTCGTGGTAGGGTT	CTGAGTCCGCCAATGTCT	184
BnC08Y1432	AAACCGAGAACTTCGTATC	CCACTATCGAGCATGACTA	213
BnC08Y1441	CGGATGGACTTTCTCGTT	GCACCTGATAGTTGGGATTA	186
BnC08Y1449	TGGGCTTCTGGTCCTAAT	GGCTAACGTGCGTATCAA	238
BnC08Y1487	TCAACTTCAAGTGTAGGGTA	GAAAGCCAAATACGAGTG	218
BnC08Y1571	TCTTTCCGACGTTGCTTT	CCACCCGTTGACAATACC	186
BnC08Y1577	GCTTGGGCTTGTGAACAG	ACCTTGGGCAGATGGAAT	194
BnC08Y1582	TGCTGAAATCACTGAGGGTA	TCTGCTTCCGACAACTGG	230
BnC08Y1610	ATAGGCTCGTAGTCTTTCCC	CATTCAAAGTGTTGTGCTCC	264
BnC08Y1595	CTGTTTAGGGACAAGTGTT	TCATTACCGATGTGGGAC	268
BnC08Y1603	TAGCTGCTTCTAGTGACCC	GATAGCCAGGAAACCATT	249
BnC08Y1606	GGTTTCCTGGCTATCATCC	GTGCTCGGTAATGGTGGA	219
BnC08Y1610	ATTCCTTTCTTCACTCCACCAC	TAGCACGGGCGGAAATAC	241
